# Synergetic Effect of rHDL and LXR Agonist on Reduction of Atherosclerosis in Mice

**DOI:** 10.3389/fphar.2020.513031

**Published:** 2020-12-16

**Authors:** Emily E. Morin, Yanhong Guo, Hongliang He, Wenmin Yuan, Whitney N. Souery, Maria V. Fawaz, Yuqing Eugene Chen, Anna Schwendeman

**Affiliations:** ^1^ Department of Pharmaceutical Sciences, University of Michigan, Ann Arbor, MI, United States; ^2^ Department of Internal Medicine*,* University of Michigan, NCRC, Ann Arbor, MI, United States; ^3^ Department of Medicinal Chemistry, University of Michigan, Ann Arbor, MI, United States; ^4^ Biointerfaces Institute, University of Michigan, Ann Arbor, MI, United States

**Keywords:** high-density lipoprotein, liver X receptor, cholesterol efflux, coronary artery disease, atherosclerosis

## Abstract

High-density lipoproteins (HDLs) are unique in that they play an important role in the reverse cholesterol transport process. However, reconstituted HDL (rHDL) infusions have demonstrated limited beneficial effect in clinical practice. This is perhaps a consequence of the limited cholesterol efflux abilities of atheroma macrophages due to decreased expression of cholesterol transporters in advanced atheromas and following rHDL infusion treatment. Thus, we propose that a combination therapy of rHDL and a liver X receptor (LXR) agonist could maximize the therapeutic benefit of rHDL by upregulating ATP-binding cassette transporters A-1 (ABCA1) and ATP-binding cassette transporter G-1 (ABCG1), and enhancing cholesterol efflux to rHDL. In macrophages, rHDL downregulated the expression of ABCA1/G1 in a dose- and rHDL composition-dependent manner. Although LXR agonist, T0901317 (T1317), upregulated the expression of ABCA1 and ABCG1, the drug itself did not have any effect on cholesterol efflux (6.6 ± 0.5%) while the combination of rHDL and T1317 exhibited enhanced cholesterol efflux from [^3^H]-cholesterol loaded J774A.1 macrophages (23.3 ± 1.3%). Treatment with rHDL + T1317 significantly reduced the area of aortic plaque in ApoE^−/−^ mice compared to PBS treated control animals (24.16 ± 1.42% vs. 31.59 ± 1.93%, *p* < 0.001), while neither rHDL nor T1317 treatment alone had a significant effect. Together, we show that rHDL paired with an LXR agonist can induce a synergetic effect in reducing atheroma burden. This synergy could lead to lower overall effective dose for both drugs, potentially overcoming the existing barriers in clinical development and renewing pharmaceutical interest in these two drug classes.

## Introduction

Atherosclerotic plaques are characterized by the accumulation of abnormal amounts of cholesterol in the artery wall. Reverse cholesterol transport (RCT) is a protective mechanism by which the body removes excess cholesterol from peripheral tissues, including the atherosclerotic plaques. HDL plays a primary role in all stages of RCT: 1) as cholesterol acceptors for cholesterol efflux from macrophages; 2) remodeling and delivery of cholesterol to the liver; and 3) releases cholesterol to the liver for the final excretion into bile and feces. Recent population studies established the inverse relationship between cholesterol efflux capacity and risk of coronary artery disease (CAD) (Khera et al., 2011; Rohatgi et al., 2014), suggesting that enhancing this process may represent a promising strategy to reduce atherosclerotic plaque burden and subsequent cardiovascular events.

Reconstituted HDL (rHDL), such as CSL-112, ETC-216 and CER-001, is an intriguing and controversial class of drugs in clinical development for treatment of CAD (Tardif et al., 2009), stemming from decades of evidence that rHDL and HDL-mimicking nanoparticles are capable of reducing atherosclerosis burden in animal models (Badimon et al., 1990; Shaw et al., 2008; Amar et al., 2010; Bielicki et al., 2010; Di Bartolo et al., 2011; Duivenvoorden et al., 2014; Sanchez-Gaytan et al., 2015; Son et al., 2016). Designed to mimic the atheroprotective function of endogenous pre-β HDL particles, rHDL is composed of apolipoprotein A-I (ApoA-I) or ApoA-I mimetic peptides complexed with phospholipids to form 8–10 nm nanodiscs (Kuai et al., 2016). They are shown to be capable of rapid mobilization of cholesterol from periphery to plasma after systemic dosing and well tolerated in patients. Intravascular ultrasound imaging studies shown the ability of 4–5 infusions of rHDL to reduce atheroma volume in CAD patients (Khan et al., 2002; Nissen et al., 2003; Miles et al., 2004; Tardif et al., 2007; Tardif et al., 2014). Based on these encouraging data, a 17,400-patient phase three trial (AEGIS-II) was initiated for CSL-112 to show possible reduction of major adverse cardiovascular events in subjects with acute coronary syndrome (Krause and Remaley, 2013; CSL Behring, 2018). Although a dose-dependent increase in plasma cholesterol efflux capacity in patients with stable atherosclerotic disease was observed following administration of CSL-112 (Gille et al., 2014; Gille et al., 2018), there was no clear dose-response relationship for rHDL infusions, with similar plaque reduction at low and high doses for ETC-216 (Nissen et al., 2003) and no atheroma regression for high doses of CER-001 (Tardif et al., 2014). This lack of dose-response may be a result of decreased expression of ATP-binding cassette transporter A1 and G1 (ABCA1 and ABCG1) following CER-001 rHDL treatment (Tardy et al., 2015).

The atheroprotective properties of endogenous HDL are largely reliant on its ability to efflux cholesterol from ABCA1/G1 transporters on the plasma membrane of atheroma foam cells and subsequently shuttle excess cholesterol to the liver for excretion (Remaley et al., 2008). Yet, under pathological CAD conditions, ABCA1 protein levels in atheroma are often reduced (Albrecht et al., 2004) and the ability of endogenous HDL to efflux cholesterol is impaired (Huang et al., 2014; Rosenson et al., 2015; Vaisar et al., 2015). rHDL infusions are designed to increase concentrations of functional cholesterol acceptors in the plasma, however they have been shown to further reduce ABCA1/G1 (Schmitz et al., 1999; Tardy et al., 2015). Decreased expression of ABCA1 has also been observed in advance plaques with administration of statins (Zanotti et al., 2006; Wong et al., 2008). ABCA1/G1 expression is regulated at the transcriptional level by liver X receptor (LXR), and several orally active small molecule LXR agonists were developed for treatment of CAD, including T0901317 (Tularik) (Katz et al., 2009; Hong and Tontonoz, 2014). For therapeutic efficacy, LXR agonists rely on endogenous HDL to serve as a cholesterol acceptors, although HDL levels and functionality are both reduced in CAD patients (Khera et al., 2011; Rohatgi et al., 2014). Clinical development of this drug class is also hindered by significant off-target toxicity (Katz et al., 2009; Kirchgessner et al., 2016), owing largely to activity of the LXRα isoform in the liver (Oosterveer et al., 2010), inducing hepatic lipogenesis and hypertriglyceridemia via activation of sterol regulatory element-binding protein-1 gene (SREBP-1), fatty acid synthase (FASN) and Cytochrome P450 Family 7 Subfamily A Member 1 (CYP7A1) (Schultz et al., 2000; Im and Osborne, 2011). Efforts are currently underway to develop agonists more specific to the LXRβ isoform to reduce toxicity seen with first-generation dual agonists (Kirchgessner et al., 2015; Kick et al., 2016; Kirchgessner et al., 2016; Zheng et al., 2016). Yet, the issue of reduced and/or dysfunctional HDL acceptors in CAD patients still limits the utility of this drug class. Hence, co-administration of rHDL and LXR agonist could serve as a synergetic treatment for atherosclerosis by addressing both ABCA1/G1 expression and cholesterol acceptor levels.

In this study, we propose to investigate the potential synergetic effect of co-administering rHDL and LXR agonist on atherosclerosis inhibition. We hypothesize that a potential synergy between two drugs stems from the abilities of 1) LXR agonists to increase expression of key cholesterol transports ABCA1 and ABCG1 in foam cells, 2) rHDL to act as a functional cholesterol acceptor, taking up excess cholesterol via interactions with ABCA1/G1 and 3) LXR agonist to overcome rHDL-induced reduction of ABCA1/G1 expression. Furthermore, a synergetic effect could potentially reduce the dose of rHDL and LXR agonist required for therapeutic efficacy, leading to a reduction in unwanted side effects and a renewed interest in clinical development of these two drug classes.

## Materials and Methods

### Reagents

22A peptide (PVLDLFRELLNELLEALKQKLK) was synthesized by Genscript (Piscataway, NJ) and purity was determined to be >95% by HPLC. Egg sphingomyelin (SM), 1,2-dipalmitoyl-sn-glycero-3-phosphocholine (DPPC), and 1-palmitoyl-2-oleoyl-*sn*-glycero-3-phosphocholine (POPC) were purchased from Avanti Polar Lipids (Alabaster, AL) and Nippon Oil and Fat (Osaka, Japan). T0901317 (T1317), was purchased from Cayman Chemical (Ann Arbor, MI). Anti-ABCA1 (ab18180) mouse monoclonal antibody and secondary antibody goat-anti-mouse IgG H + L (HRP) (ab205719) were purchased from Abcam (Cambridge, United Kingdom). Anti-beta-ACTIN rabbit antibody (#4970) and HRP-conjugated goat anti-rabbit secondary antibody (#7074) were purchased from Cell Signaling Technology (Danvers, MA).

### Reconstituted High-Density Lipoprotein Preparation

Discoidal ETC-642 mimicking rHDL was made by co-lyophilization followed by thermal cycling, as previously described (Di Bartolo et al., 2011; Schwendeman et al., 2015). Briefly, 22A peptide, SM and DPPC were dissolved in glacial acetic acid at a weight ratio of 1:1:1. The resulting solution was frozen and freeze-dried overnight. The lyophilized powder was reconstituted in PBS. The resulting suspension was subjected to heat/cool cycles above and below the lipid T_m_ (5 min heating at 55°C and 5 min cooling at room temperature) until a clear solution of rHDL nanoparticles was formed. The rHDL solution was adjusted to pH 7.4 and 0.2 µm sterile filtered.

ApoA-I containing rHDLs were prepared by a thin-film method, with protein and lipid compositions similar to those reported for clinically tested rHDLs (Shaw et al., 2008; Andrews et al., 2017). Briefly, lipids (SM or POPC) were dissolved in chloroform and dried with nitrogen gas. The resulting lipid film was hydrated by a solution of ApoA-I to achieve the desired weight ratio of ApoA-I:lipids (1:1.5 ApoA-I:POPC for “CSL-112 like,” and 1:2.7 ApoA-I:SM for “CER-001 like”). The resulting mixture was vortexed, followed by brief (<30 s) bath sonication in order to fully disperse the lipid film. The solution then underwent three heat/cool cycles, as described above, to form a clear, homogeneous solution of rHDL and sterilized by 0.22 µm filtration. All rHDL concentrations and dosages are expressed in terms of 22A peptide or ApoA-1 protein concentration.

Lipid emulsion controls were prepared by dissolving lipids (SM, DPPC, or POPC) in chloroform and dried with nitrogen gas. The resulting lipid films were rehydrated in PBS, vortexed, briefly sonicated (<30 s), and subject to three heat/cool cycles above and below the lipid transition temperature.

### Reconstituted High-Density Lipoprotein Characterization

Quality of rHDL particles was assessed using the following analytical techniques, and is reported in (Supplementary Table SIII). Size distribution was determined by dynamic light scattering on a Malvern Zetasizer Nano ZSP (Westborough, MA), and particle purity was determined by gel permeation chromatography with UV detection at 220 using a Tosoh TSK gel G3000SWxl column (Tosoh Bioscience, King of Prussia, PA) on a Waters HPLC (Schwendeman et al., 2015).

### Cell Culture

J774A.1 macrophages were purchased from ATCC (Manassas, VA) and maintained in Dulbecco’s Modified Eagle’s Medium containing 10% fetal bovine serum (FBS) and 100 U/mL penicillin/streptomycin.

### Western Blot

Cells were switched to DMEM containing 1% FBS overnight prior to treatment. Then, cells were treated with rHDL (100 μg/ml), T1317 (1 μM), PBS, or rHDL + T1317 for 24 h. After treatment cells were washed twice with ice cold PBS and lyzed in radioimmune precipitation assay buffer containing cOmplete^™^ mini, EDTA-free protease inhibitor cocktail (Sigma). Protein lysates (30 μg/lane) were separated by 7.5% SDS-polyacrylimide gel and transferred to 0.45 μm PVDF membrane using a Bio-Rad semi-dry transfer system. ABCA1 expression was determined by Western Blotting using antibodies for ABCA1 (1:2,000) and beta-ACTIN (1:1,000). Signal was detected using horseradish peroxidase-conguated secondary antibodies (Cell Signaling Technologies) and ECL (Amersham Biosciences).

### Cholesterol Efflux

Cholesterol efflux studies were performed, as described previously (Guo et al., 2015; Schwendeman et al., 2015). J774.1 macrophages (ATCC, Manassas, VA) were labeled with 1 μCi/ml [1,2-^3^H]-cholesterol (Perkin Elmer, United States) in Dulbecco’s Modified Eagle’s Medium containing 0.5% fatty acid-free bovine serum albumin (BSA) and 5 μg/ml ACAT inhibitor Sandoz 58-035 (Sigma) for 24 h. Cells were then washed twice with PBS and equilibrated for 24 h in DMEM containing 0.5% BSA. Then cells were washed and treated with PBS, rHDL (50 μg/ml), T1317 (1 μM), or rHDL (50 μg/ml) + T1317 (1 μM) for 12 h. Medium was collected and cells were lyzed and collected. Efflux in cellular and media fractions was quantified by liquid scintillation and expressed as a percentage of total cell [^3^H]-cholesterol content. Nonspecific efflux to the media is reflected in the PBS control groups.

### Quantitative Real-Time PCR

Cells were switched to DMEM containing 1% FBS overnight prior to treatment. Then, cells were treated with rHDL (1, 10, or 100 μg/ml based on ApoA-I or 22A peptide), T1317 (1 μM), PBS or rHDL + T1317 for 24 h. At indicated time points post-rHDL treatment, cells were washed twice with cold PBS and scraped and collected in lysis buffer. Total RNA was purified with the GeneJET RNA Purification Kit (Thermo Scientific), followed by cDNA synthesis with the SuperScript III First-Strand Synthesis System kit (Invitrogen). For mouse tissue, livers or aortas were homogenized and RNA isolated using TRIzol^®^ reagent (ThermoFisher). qPCR was carried out using Power SYBR Green Master Mix (Applied Biosystems) and primers for mouse *Abca1*, *Abcg1, Fasn, and Srebp1* (Supplementary Table SII). Relative abundance of mRNA was normalized to the geometric average of housekeeping controls *18sRNA* and *Ppia*.

### Anti-Atherosclerosis Study in ApoE^−/−^ Mice

ApoE^−/−^ male mice were purchased from Jackson Laboratories (Bar Harbor, ME) and housed at 22 ± 1°C in a 12:12-h light-dark cycle. All animal work was performed in accordance with the guidelines set by the University of Michigan Animal Care and Use Committee. ApoE^−/−^ mice (7–9 weeks old) were placed on a high-fat/high-cholesterol diet (HFHC, 21% fat, 34% sucrose, and 0.2% cholesterol, Harlan, TD. 88137) for 7 weeks prior to dosing, and were maintained on the HFHC diet for the duration of the study. Mice were randomly assigned into groups (n = 8/group) to receive either vehicle (PBS), rHDL (30 mg/kg), T1317 (1.5 mg/kg) or rHDL (30 mg/kg) + T1317 (1.5 mg/kg) three times per week for 6 weeks by I.P. injection. 48 h following the final dose, mice were sacrificed and perfused with 10% normal buffered formalin to fix tissues. Aorta roots were micro-dissected, embedded in OCT, and frozen in liquid nitrogen for analysis of lesion area. The atherosclerotic lesions in the aortic sinus were analyzed in three different regions, each separated by 80 μm. Lesions were visualized by Oil Red O staining and lesion area quantified using ImageJ software. Livers and aortas were collected and flash frozen for use in downstream PCR applications. Plasma was collected following a terminal blood draw and analyzed for total cholesterol (TC), triglycerides (TG), HDL-C, LDL-C, alanine transaminase (ALT), and aspartate transaminase (AST) by the University of Michigan Clinical Chemistry Core.

### Statistical Analysis

All experiments were performed in triplicate. Data are presented as means ± SEM. Statistical significance between groups was assessed by one-way ANOVA with Tukey’s correction for multiple comparisons and *p* < 0.05 were considered significant. All groups were determined to be normally distributed by Shapiro-Wilk and Kolmogorov-Smirinov normality tests (*p* < 0.05).

## Results

### Reconstituted High-Density Lipoprotein Affects ATP-Binding Cassette Transporter A-1 and ATP-Binding Cassette Transporter G-1 Gene Expression *in vitro*

**FIGURE 1:**
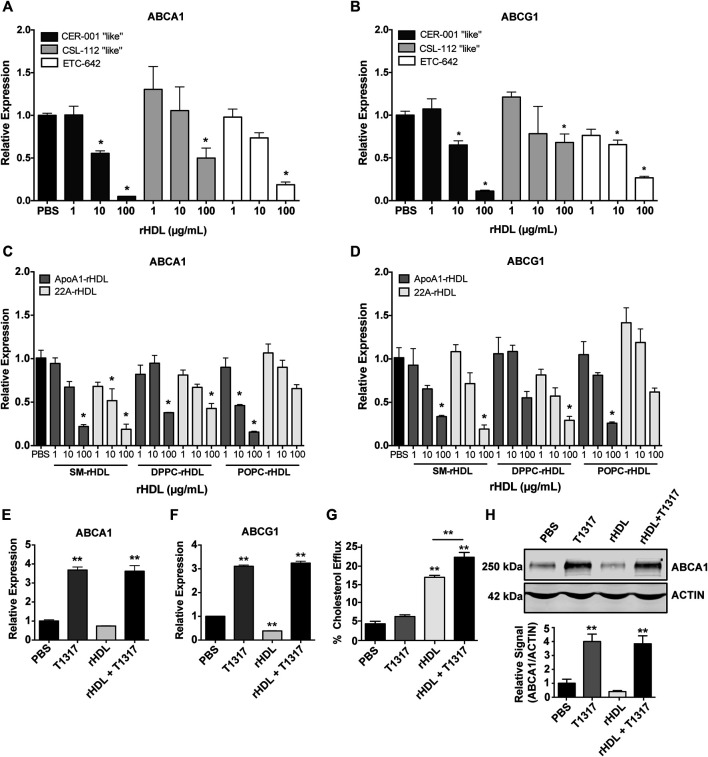
Effect of reconstituted high-density lipoprotein (rHDL) on ATP-binding cassette transporter A-1 (ABCA1) and ATP-binding cassette transporter G-1 (ABCG1) expression and rescue by LXR liver X receptor (LXR) agonists *in vitro* and *in vivo*. J774A.1 murine macrophages treated for 24 h with clinically-mimicking rHDLs CER-001 [apolipoprotein A-I (ApoA-I)-sphingomyelin (SM) 1:2.7 w/w], CSL-112 [ApoA-I-1-palmitoyl-2-oleoyl-*sn*-glycero-3-phosphocholine (POPC) 1:1.5 w/w], or ETC-642 [22A-1,2-dipalmitoyl-sn-glycero-3-phosphocholine (DPPC)-SM 1:1:1 w/w] exhibit decreased levels of ABCA1 **(A)** and ABCG1 **(B)** mRNA relative to PBS treated controls, as measured by RT-qPCR. Effect of rHDL composition on ABCA1 **(C)** and ABCG1 **(D)** expression was probed by incubating J774A.1 cells for 24 h with rHDL prepared from either 22A peptide or ApoA-I protein complexed with SM, DPPC, or POPC lipids (all at 1:2 protein:lipid ratio by weight). Effect of adding LXR agonist on ABCA1 **(E)** and ABCG1 **(F)** expression in macrophages treated with different combinations of rHDL (100 μg/ml) or T1317 (1 µM) for 24 h). [^3^H]-cholesterol efflux to sHDL acceptor (50 μg/ml) in J774A.1 cells incubated with or without T1317 (1 µM) for 24 h **(G)**. ABCA1 protein levels in J774A.1 macrophages were determined by Western Blot following 24 h incubation rHDL (100 μg/ml 22A) in the presence/absence of T1317 (1 µM) **(H)**. rHDL concentration is expressed in terms of peptide or protein. Data represented as mean ± SEM. *p < 0.05, **p < 0.01 relative to PBS control or as otherwise indicated.

As rHDL composition is known to significantly affect its cholesterol efflux capacity (Davidson et al., 1995; Schwendeman et al., 2015) and possibly ABCA1/ABCG1 expression, we prepared rHDL mimicking three clinically tested products, i.e. CER-001, CSL-111 and ETC-642 to obtain clinically relevant data. The rHDL formulations varied in both lipid and protein/peptide composition: CER-001 (ApoA-I:SM, 1:2.7 w/w), CSL-112 (ApoA-I:POPC, 1:1.5 w/w), and ETC-642 (22A:DPPC:SM, 1:1:1 w/w/w). Using J774A.1 macrophages treated for 24 h with rHDL, we show that increasing concentrations of rHDL decreased mRNA expression of both ABCA1 and ABCG1 relative to PBS (Figures 1A,B). We also show that rHDL composition affects the magnitude of reduction for ABCA1 expression, with 100 μg/ml treatment reducing ABCA1 expression >85% for CER-001 “like” rHDL, >60% for ETC-642, >45% for CSL-112 “like” rHDL (Figure 1A). A similar trend was observed for ABCG1 expression (Figure 1B).

In attempt to identify the driving component of rHDL responsible for downregulation of ABCA1/G1 mRNA, we incubated macrophages for 24 h with either 22A peptide, ApoA-I protein, or lipid emulsions of SM, DPPC, or POPC. Neither 22A peptide nor ApoA-I protein induced significant changes in ABCA1 or ABCG1 (Supplementary Figures SIA,B), indicating that rHDL lipid composition may have a greater influence on gene expression. Consistent with previous reports (Langmann et al., 1999; Akopian et al., 2015), we noticed significant reductions in macrophages treated with lipids alone, with slightly stronger responses elicited for SM and DPPC compared to POPC (Supplementary Figures SIC,D). However, reduction in ABCA1 and ABCG1 mRNA were higher overall for rHDL particles rather than any individual component alone.

We then investigated how whole rHDL particles prepared from either 22A peptide or ApoA-I and lipids SM, DPPC, or POPC influenced ABCA1 and ABCG1 mRNA expression in the same *in vitro* system. In this case, rHDL was prepared similarly for both 22A and ApoA-I based formulations, holding the peptide/protein to lipid ratio at 1:2 by weight for all formulations. Similar to the results seen for our clinically-mimicking rHDLs, both ApoA-I and 22A-based rHDL at 100 μg/ml were able to reduce relative expression of ABCA1 and ABCG1, with no obvious differences between 22A vs. ApoA-I or SM vs. DPPC vs. POPC under the tested conditions (Figures 1C,D).

### Reconstituted High-Density Lipoprotein-Liver X Receptor Agonist Co-Administration Synergistically Increases ATP-Binding Cassette Transporter A-1 and ATP-Binding Cassette Transporter G-1 Expression and Increases Cholesterol Efflux

Given our hypothesis that co-administration of rHDL and an LXR agonist could act in synergy to increase cholesterol transporter expression and maximize cholesterol efflux, we decided to test the LXR agonist, T1317, *in vitro* for its ability to upregulate ABCA1 and ABCG1 expression in the presence of rHDL. We chose ETC-642, a peptide-based rHDL, as our model rHDL at a concentration of 100 μg/ml, as this concentration exhibited maximal inhibition of ABCA1 and ABCG1 expression in the earlier experiments. J774A.1 macrophages were treated for 24 h with PBS, rHDL (100 μg/ml) T1317 (1 μM), or rHDL + T1317 at 100 μg/ml and 1 μM, respectively. As seen in Figure 1E, the combination of rHDL and T1317 increased ABCA1 mRNA expression >3-fold compared to PBS-treated controls (*p* < 0.001). This trend also held true for ABCG1 expression (Figure 1F). Notably, the combination of rHDL and T1317 overcame rHDL-induced downregulation and even increased expression levels of ABCA1/G1 in a manner similar to T1317 alone (Supplementary Figure SII).

To examine the effect of combination treatment on ABCA1 protein levels, we treated J774A.1 macrophages for 24 h with PBS, rHDL (100 μg/ml), T1317 (1 μM), or rHDL + T1317 (100 μg/ml and 1 μM, respectively) and performed Western Blot. Aligning with our previous data, treatment with rHDL alone diminished ABCA1 protein levels, while the addition of T1317 restored protein levels to that similar to PBS and free T1317-treated controls (Figure 1H). The addition of T1317 also led to an increase in TC efflux over rHDL alone (23.3 ± 1.3% vs. 17.6 ± 0.5%, respectively), while T1317 alone (6.6 ± 0.5%) had little measurable efflux (Figure 1G).

### Reconstituted High-Density Lipoprotein/T1317 Combination Therapy Inhibits Atherosclerosis Progression *in vivo*

**FIGURE 2:**
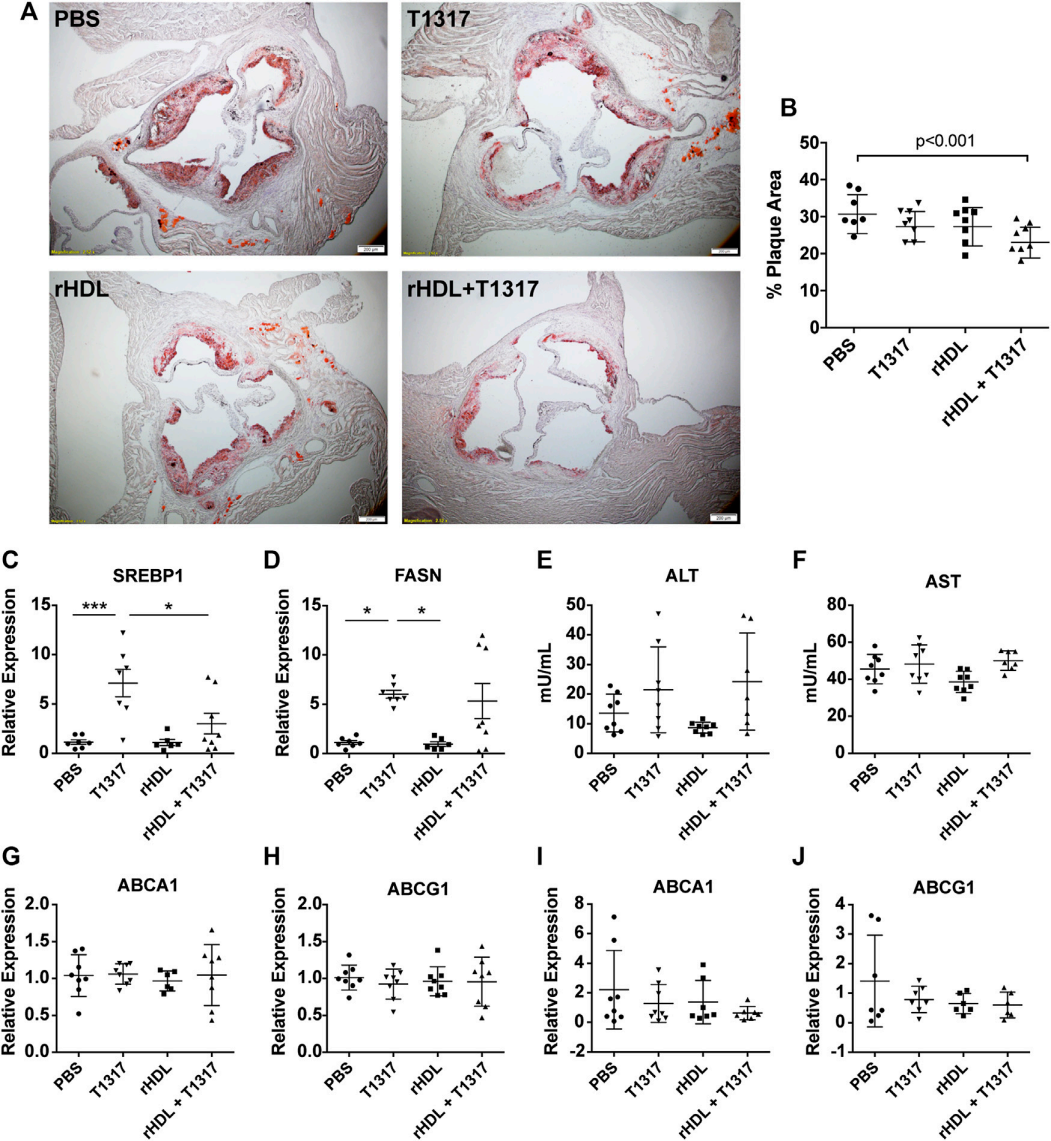
Reconstituted high-density lipoprotein (rHDL)/T1317 inhibition of atherosclerosis progression in ApoE^−/−^ mice. ApoE ^−/−^ mice were fed a high fat/high cholesterol diet to induce atherosclerosis prior to treatement. At week 7, animals were dosed by I.P. injection three times weekly for a duration of 6 weeks with PBS, rHDL (30 mg/kg), rHDL + T1317 (30 mg/kg rHDL and 1.5 mg/kg T1317), or T1317 (1.5 mg/kg). At the end of treatment mice were sacrificed and aortas were fixed and sectioned for plaque area visualization by Oil Red O staining **(A)**. Plaque area was quantified using ImageJ **(B)**. Liver safety biomarkers in ApoE^−/−^ mice following 6 weeks of anti-atherosclerosis therapy with PBS, rHDL (30 mg/kg), rHDL + T1317 (30 mg/kg + 1.5 mg/kg, respectively), or T1317 (1.5 mg/kg) were measured. Gene expression of sterol response element binding protein 1 and fatty acid synthase were determined in liver lysates **(C,D)** and activity of liver enzymes alanine aminotransferase and aspartate aminotransferase were determined in the plasma **(E,F)**. ATP-binding cassette transporter A-1 (ABCA1) and ATP-binding cassette transporter G-1 (ABCG1) expression were measured in the aortas **(G,H)** and livers **(I,J)** 48 h after administration of the final treatment dose. Data are presented as mean ± SEM. *p < 0.05, ***p < 0.001.

While our findings *in vitro* suggest that rHDL + T1317 combination therapy can upregulate ABCA1 and ABCG1 at the mRNA and protein level as well as increase TC efflux when compared to either drug alone, we wanted to confirm whether these findings confer a greater anti-atherosclerosis effect *in vivo*. To do this, ApoE^−/−^ mice were fed a HFHC diet for 7 weeks prior to the start of dosing in order to establish atherosclerosis. Then, mice were randomized to receive PBS, rHDL (30 mg/kg), T1317 (1.5 mg/kg), or rHDL + T1317 combination therapy (injected into two sites with rHDL at 30 mg/kg and T1317 at 1.5 mg/kg, respectively) via i.p. injection 3×/week for 6 weeks while maintained on the HFHC diet. Mice receiving rHDL + T1317 combination therapy had a marked reduction in total plaque area compared to PBS controls (24.16 ± 1.42% vs. 31.59 ± 1.93%, respectively), assessed by Oil Red O staining at the aortic root (Figures 2A,B). There were no significant changes in plaque volume for either rHDL (28.30 ± 1.78%) or free T1317 (28.30 ± 1.39%) groups compared to PBS-treated controls, supporting our hypothesis that rHDL + T1317 combination therapy could act in synergy to maximize the anti-atherosclerosis effect over either drug individually. There were no measurable differences in aorta or liver ABCA1 or ABCG1 mRNA expression between any of the groups (Figures 2G–J), as the final analyses were done 48 h after the final dose.

In addition to ABCA1 and ABCG1, LXR also regulates transcription of cholesterol biogenesis genes, and the associated side-effects have hindered LXR agonist clinical progress. We found T1317 treatment significantly increased the expression of FASN and sterol-response-element binding protein 1 (Srebp1). Mice receiving rHDL + T1317 combination therapy had slightly reduced expression of Srebp1 compared to T1317-only treated mice (*p* < 0.05) (Figures. 2C,D) but were still elevated compared to PBS and rHDL only treated groups. No significant differences in ALT or AST activity between any of treatment groups (Figures 2E,F). There were no significant differences in plasma lipids (TC, HDL-C, LDL-C, TG) between any of the treatment groups likely because they were measured 48 post-dose, after rHDL-mobilized cholesterol is eliminated (Supplementary Table SI).

## Discussion

Numerous studies have demonstrated the safety various rHDL particles and their abilities to alter atherosclerotic plaque composition and decrease the overall plaque burden (Nissen et al., 2003; Tardif et al., 2007; Di Bartolo et al., 2011; Keyserling et al., 2011), yet, there are many potential reasons why rHDL-infusion therapies failed to show efficacy in larger phase two trials (Tardif et al., 2014; Nicholls et al., 2018). In a likely attempt for the cell to limit further cholesterol loss, decreased expression of ABCA1/G1 transporters after high dose rHDL infusions is observed. However, this obstructs the first step of RCT, leading to reduced rHDL-mediated cholesterol efflux from foam cells in the atherosclerotic plaques upon subsequent dosing. ABCA1 and ABCG1 are critical enablers of rHDL’s anti-atherosclerotic function. Here, we show for the first time a synergy between rHDL and LXR agonist therapies, stemming from the dual effects: 1) LXR agonist increases cholesterol transporters expression and cholesterol efflux in macrophages; 2) rHDL works as cholesterol acceptor. Combined therapy will make up for rHDL-triggered downregulation of cholesterol transporters in macrophages, allowing subsequent rHDL infusions to achieve maximal efficacy.

Infusion of CER-001 did not promote regression of coronary atherosclerosis in statin-treated patients with ACS and high plaque burden (Nicholls et al., 2018), which may be due to the strong (50%) downregulation of ABCA1 mRNA and membrane protein expression at higher doses of CER-001 (Tardy et al., 2015). In this study, we first compared the effects of clinically relevant rHDLs on the expression of ABCA1/ABCG1 and found dose-dependent down regulation of ABCA1/ABCG1 in the presence of all types of rHDL. We also observed that CER-001 showed the greatest effect on ABCA1/ABCG1 reduction, ETC-642 having slightly less, and CSL-112 having the least reduction of the three. This may be attributed to the higher cholesterol efflux capacity of these rHDLs, as SM-containing rHDL has been shown to induce greater cholesterol efflux from foam cells than POPC-based rHDLs due to its higher cholesterol binding affinity (Davidson et al., 1995; Yancey et al., 2000; Kučerka et al., 2010; Ma et al., 2012; Schwendeman et al., 2015). In addition, CER-001 has the highest lipid-to-protein ratio (2.7:1 w/w), followed by ETC-642 (2:1) and CSL-112 (1.5:1), which may also result in a higher cholesterol acceptor capacity. The fact that the downregulation of ABCA1/ABCG1 was also observed for CSL-112 is of particular importance, as this rHDL product is currently undergoing a large phase three study (AEGIS-II). Failure of this study will likely constitute the end of rHDL therapeutic development, while success will stimulate the interest in rHDL. While CSL-112-like rHDL inhibited ABCA1/ABCG1 to a lesser extent than CER-001-like in our study, the CSL-112 dose used in AEGIS-II is about 10-fold higher than the CER-001 dose that failed to show efficacy in CHI SQUARE trial (Michael Gibson et al., 2016; Nicholls et al., 2018). Thus, understanding how composition of rHDL impacts downregulation of ABCA1/ABCG1 is of critical importance.

Clinical utility of LXR agonists has been limited due to adverse side-effects, including induction of hepatic lipogenesis and increases in circulating LDL (Schultz et al., 2000; Lee and Tontonoz, 2015). Attempts to solve this issue have emerged in the form of drug-delivery systems to lower systemic exposure. Approaches have included targeted antibody-drug conjugates (Lim et al., 2015), poly(lactic-co-glycolic acid) (PLGA) and other modified nanoparticle encapsulation (Zhang et al., 2015; Yu et al., 2017), and even ApoA-I-containing PLGA nanoparticles to target atherosclerotic plaques (Sanchez-Gaytan et al., 2015). While these approaches have been successful in increasing ABCA1 expression levels, they rely on endogenous HDL to accept the resulting effluxed cholesterol, despite impaired functionality of HDL under pathological conditions (Huang et al., 2014; Rosenson et al., 2015; Vaisar et al., 2015). We previously showed that encapsulation of T1317 in rHDL nanoparticles promotes atherosclerosis regression and avoids systemic toxicity observed with free T1317 administration (Guo et al., 2018). We hypothesize that the improved activity of T1317-sHDL nanoparticles is in part due to the ability of sHDL drug carriers to serve as cholesterol acceptors. Hence, we sought to explore the potential synergy between the rHDL drug carrier and LXR agonist administered separately, leading to the current study.

We see *in vitro* that the combination of rHDL and LXR agonist can upregulate ABCA1/ABCG1 mRNA and protein and enhance overall cholesterol efflux. In a long-term dosing study in atherosclerosis mice, we witnessed a reduction in overall plaque area in mice treated with both rHDL and T1317 compared to non-treated mice, and this effect was not seen for animals treated with low doses of rHDL or T1317 only. In this study, we were able to achieve atheroma reduction using a 1.5 mg/kg dose of T1317 that was administered only 3 times/week for 6-weeks. In comparison, studies administering free T1317 by oral gavage required daily dosing in the range of 10–50 mg/kg to see significant atheroma reduction, but at the cost of unwanted toxicity (Dai et al., 2008; Ou et al., 2008; Honzumi et al., 2011; Kappus et al., 2014). And while we did observe a slight reduction in liver Srebp1 mRNA levels in the combination therapy group, more significant reductions in T1317-induced toxicity will likely be seen with further dose reduction following more extensive pharmacological dose-response studies.

In conclusion, we show that rHDL and LXR agonists can act in synergy leading to increased anti-atherosclerosis efficacy when administered together as opposed to individually. Thus, co-administration of rHDL and LXR agonists could be of great potential interest to the pharmaceutical industry and revive interest in development of these two classes of drugs.

## Data Availability Statement

The datasets generated for this study are available on request to the corresponding author.

## Ethics Statement

The animal study was reviewed and approved by University of Michigan Animal Care and Use Committee.

## Author Contributions

EM performed all experiments with crucial help from YG, HH, WY, WS and MF. AS and EC conceived and designed the research. EM wrote the manuscript. AS, YG and EC helped with data interpretation and edited the manuscript.

## Funding

This study was supported by NIH grants HL068878 and HL134569 (YC), GM113832 (AS), HL134569 (YC and AS), NIH grants K12-GM088021, T32-HL007736, and T32-GM008353 (EM), T32-GM07767 (MF) and T32-HL125242 (EM, MF), AHA grants 15SDG24470155 (YG), 13SDG17230049 (AS) and 16POST27760002 (WY), AFPE fellowship (MF), and FCVC Summer Fellowship Program (WS).

## Conflicts of Interest

The authors declare that the research was conducted in the absence of any commercial or financial relationships that could be construed as a potential conflict of interest.

## Abbreviations

ABCA1, ATP-binding cassette transporter A-1; ABCG1, ATP-binding cassette transporter G-1; ApoA-I, apolipoprotein A-I; CAD, coronary artery disease; HDL, high-density lipoprotein; LXR, liver X receptor; rHDL, reconstituted high-density lipoprotein; RCT, reverse cholesterol transport; DPPC, 1,2-dipalmitoyl-sn-glycero-3-phosphocholine; POPC, 1-palmitoyl-2-oleoyl-*sn*-glycero-3-phosphocholine; SM, sphingomyelin.
