# Supplementary material for: Synergetic Effect of rHDL and LXR Agonist on Reduction of Atherosclerosis in Mice
Source: Front Pharmacol. 2020 Dec 16;11:513031. doi: 10.3389/fphar.2020.513031 (PMC7772318; doi:10.3389/fphar.2020.513031)
Supplement: Supplementary file 1 [file datasheet1.pdf]

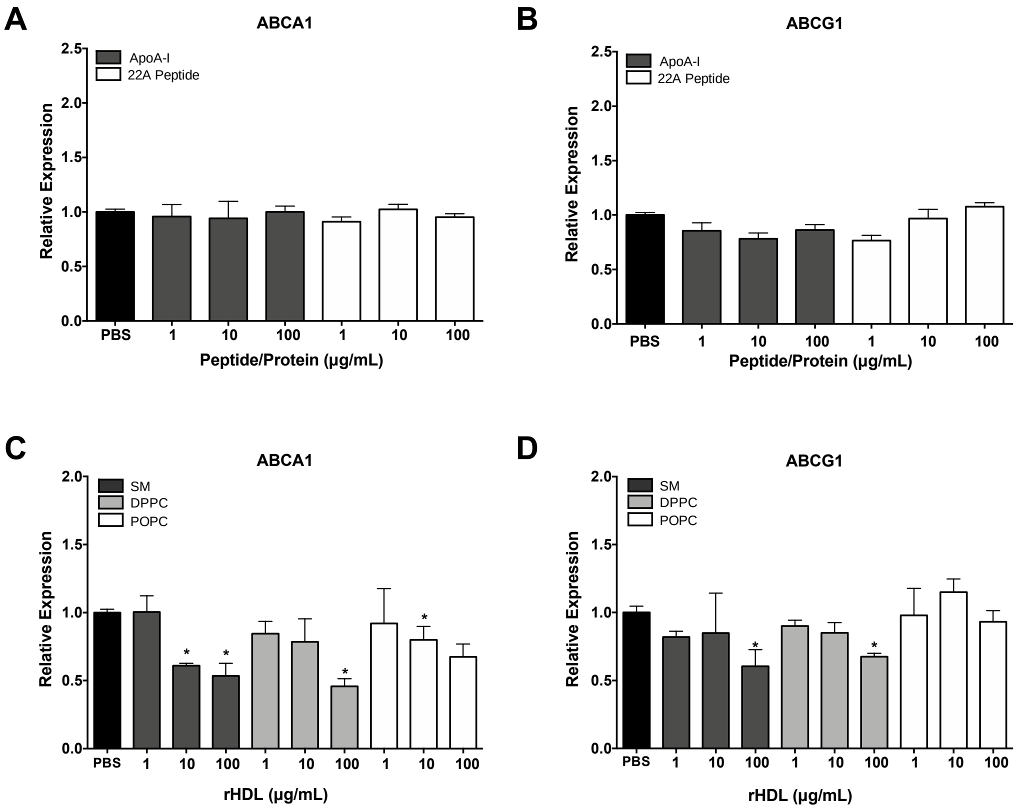

Figure SI. Effect of rHDL individual components on ABCA1 and ABCG1 expression in vitro. ABCA1 and ABCG1 expression in J774A.1 murine macrophages treated for 24 h with 22A peptide or ApoA-I protein (**A-B**) or lipid emulsions of SM, DPPC, or POPC (**C-D**). Expression values were determined relative to PBS treated controls. Data represented as mean ± SEM. *p<0.05

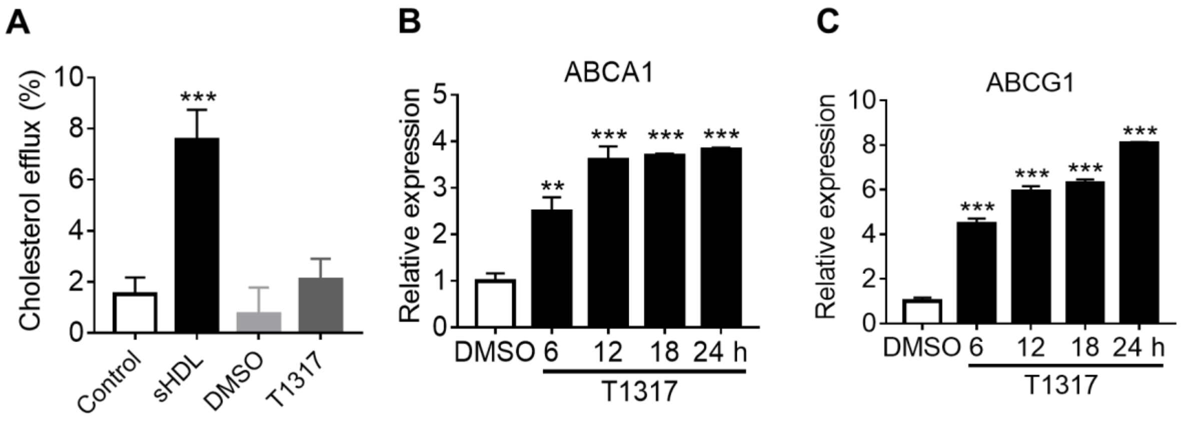

Figure SII. Effect of T1317 on cholesterol efflux and time-dependent ABCA1 and ABCG1 mRNA expression in vitro. Murine J774.1 macrophages (ATCC, Manassas, VA) were seeded in 48-well plates for 24 h. Cells were labeled with 1 µCi/ml [1,2-^{3}H]-cholesterol (Perkin Elmer, USA) in DMEM containing 0.5% fatty acid-free bovine serum albumin (BSA) and 5 µg/ml ACAT inhibitor Sandoz 58–035 (Sigma) overnight. Then cells were washed twice with PBS and equilibrated for 24 h in DMEM containing 0.5% BSA. Cholesterol efflux was measured as the percentage of [^{3}H]-cholesterol in the medium after 5 h of incubation with sHDL at 50 µg/ml, DMSO or T1317 at 0.1 µM (n = 3 per group) **(A)**. THP-1 cell derived macrophages were treated with DMSO or T1317 at 0.1 µm for indicated time points. The expression of ABCA1 **(B)** and ABCG1 (C) were determined by qRT-PCR (n=3). Data were analyzed by two-way ANOVA with Dunnett's multiple comparisons test. ***P<0.01 compared to control.

Table SI. Plasma lipid markers. Total cholesterol (TC), HDL-cholesterol (HDL-C), LDL-cholesterol (LDL-C), and triglycerides (TG) were measured in plasma collected from $ApoE^{-/-}$ fed a high fat/high cholesterol diet for 6 weeks followed by treatment with PBS, rHDL (30 mg/kg), rHDL+T1317 (30 mg/kg + 1.5 mg/kg, respectively), or T1317 (1.5 mg/kg) for 6 weeks. Blood was collected 48 h following the administration of the final dose. Data are expressed as mean ± SEM.

Parameter	PBS	rHDL	rHDL + T1317	T1317
N	8	8	5	6
TC (mg/dL)	1650 ± 54.9	1474 ± 64.3	1417 ± 116.1	1660 ± 131.7
HDL-C (mg/dL)	19.6 ± 1.3	22.0 ± 1.7	23.8 ± 0.8	21.83 ± 2.3
LDL-C (mg/dL)	629.4 ± 38.4	541.8 ± 27.0	488.0 ± 58.7	508.7 ± 89.2
TG (mg/dL)	228.4 ± 22.9	260.6 ± 33.3	326.4 ± 44.7	294.3 ± 32.4

Table SII. Primer sequences used for RT-PCR assays.

Gene	Forward	Reverse
**mAbca1**	gggagtcccagaaaaggaag	tgtggttggttcatccagaa
**mAbcg1**	gtaccatgacatcgctggtg	agccgtagatggacaggatg
**mFasn**	ctgaagagcctggaagatcg	gtcacacacctgggagaggt
**mSrebp1**	ggagccatggattgcacatt	gcttccagagaggaggccag
**m18sRNA**	ggaagggcaccaccaggagt	tgcagccccggacatctaag
**mPpia**	gtctccttcgagctgtttgc	gatgccaggacctgtatgct

Table SIII. Size Distribution of rHDL Particles. Size distribution for all prepared rHDL formulations was performed using dynamic light scattering (DLS) on a Malvern Zetasizer Nano ZSP. Hydrodynamic diameters are reported as z-average mean ± SD (nm). Polydispersity index (PDI) is also reported for each formulation.

rHDL	Size (nm)	PDI
**ETC-642**	10.81 ± 0.15	0.19
**CER-001 "like"**	11.05 ± 0.11	0.18
**CSL-112 "like"**	N/A	N/A
**22A-SM**	12.02 ± 0.67	0.22
**22A-DPPC**	11.02 ± 1.25	0.13
**22A-POPC**	15.66 ± 0.87	0.27
**ApoA-1-SM**	14.98 ± 0.29	0.26
**ApoA-1-DPPC**	14.42 ± 0.93	0.25
**ApoA-1-POPC**	13.30 ± 1.34	0.18
